# Real‐time analysis and display of quantitative measures to track and improve clinical workflow

**DOI:** 10.1002/acm2.13610

**Published:** 2022-08-03

**Authors:** Reshma Munbodh, Toni M. Roth, Kara L. Leonard, Robert C. Court, Utkarsh Shukla, Sarah Andrea, Marissa Gray, Gregg Leichtman, Eric E. Klein

**Affiliations:** ^1^ Department of Radiation Oncology Alpert Medical School of Brown University Providence Rhode Island USA; ^2^ Department of Radiation Oncology Columbia University Irving Medical Center New York New York USA; ^3^ Department of Radiation Oncology University of Washington in St. Louis St. Louis Missouri USA; ^4^ Department of Radiation Oncology Rhode Island Hospital Providence Rhode Island USA; ^5^ Institute for Adaptive and Neural Computation School of Informatics University of Edinburgh Edinburgh UK; ^6^ Lifespan Biostatistics Epidemiology and Research Design Core Rhode Island Hospital Providence Rhode Island USA; ^7^ OHSU‐PSU School of Public Health Portland Oregon USA; ^8^ School of Engineering Brown University Providence Rhode Island USA; ^9^ Clario Philadelphia Pennsylvania USA

**Keywords:** dashboard, database, electronic medical records, quality assurance, radiation therapy

## Abstract

**Purpose:**

Radiotherapy treatment planning is a complex process with multiple, dependent steps involving an interdisciplinary patient care team. Effective communication and real‐time tracking of resources and care path activities are key for clinical efficiency and patient safety.

**Materials and Methods:**

We designed and implemented a secure, interactive web‐based dashboard for patient care path, clinical workflow, and resource utilization management. The dashboard enables visualization of resource utilization and tracks progress in a patient's care path from the time of acquisition of the planning CT to the time of treatment in real‐time. It integrates with the departmental electronic medical records (EMR) system without the creation and maintenance of a separate database or duplication of work by clinical staff. Performance measures of workflow were calculated.

**Results:**

The dashboard implements a standardized clinical workflow and dynamically consolidates real‐time information queried from multiple tables in the EMR database over the following views: (1) CT Sims summarizes patient appointment information on the CT simulator and patient load; (2) Linac Sims summarizes patient appointment times, setup history, and notes, and patient load; (3) Task Status lists the clinical tasks associated with a treatment plan, their due date, status and ownership, and patient appointment details; (4) Documents provides the status of all documents in the patients' charts; and (5) Diagnoses and Interventions summarizes prescription information, imaging instructions and whether the plan was approved for treatment. Real‐time assessment and quantification of progress and delays in a patient's treatment start were achieved.

**Conclusions:**

This study indicates it is feasible to develop and implement a dashboard, tailored to the needs of an interdisciplinary team, which derives and integrates information from the EMR database for real‐time analysis and display of resource utilization and clinical workflow in radiation oncology. The framework developed facilitates informed, data‐driven decisions on clinical workflow management as we seek to optimize clinical efficiency and patient safety.

## 1 INTRODUCTION

Radiation treatment planning is a complex process with multiple, dependent steps conducted by an interdisciplinary care team.^1^ When a patient is admitted for external beam radiation therapy, treatment planning usually begins with the acquisition of a CT of the patient. A dosimetrist or physicist creates a highly conformal patient‐specific three‐dimensional treatment plan from the CT to deliver a physician‐prescribed dose to a physician‐delineated target volume. The plan is calculated using sophisticated treatment planning software that models the interaction of high‐energy ionizing radiation beams with tissue and consequently radiation dose deposition in the target and surrounding tissue. The integrity, safety, and deliverability of the calculated treatment plan are subsequently verified by the attending physician, medical physicists, and radiation therapists. The plan also undergoes peer review during departmental chart rounds. Finally, the plan is transferred from the treatment planning system to the electronic medical record (EMR) system, simulated and delivered to the patient on a linear accelerator in a radiation‐shielded vault by a team of radiation therapists, sometimes under the supervision of a physician or medical physicist.

Radiation treatment planning is the stage where errors are most likely to originate.^2^ From the time of CT simulation to treatment delivery on the linear accelerator, a team from at least four subspecialties in radiation oncology will have been involved at different stages in the creation and quality assurance of the treatment plan. The team will also have worked with multiple types of software and hardware, possibly from different vendors, in the process, often under stringent time constraints. Having a well‐defined and tractable clinical workflow, effective communication among a patient's care team,^3^ and care coordination through real‐time and automated tracking of resources, work allocation and completion helps ensure clinical efficiency and patient safety. However, a lack of standardization in clinical practice, inherent limitations of the EMR in displaying consolidated information that effectively communicates resource utilization and progress in the creation and delivery of the treatment plans,^4^ and a consequent lack of quantitative performance measures of workflow in radiation oncology are all challenges towards achieving these goals. These challenges are exacerbated by the need for specialized computation skills to implement solutions to extract information from the EMR.

To illustrate the fragmented and time‐consuming nature of resource utilization and patient care pathway data visualization in the departmental EMR, currently new CT simulations and new treatment start in the clinic at our institution are identified by visually inspecting the schedule for every CT simulator and linear accelerator, each in a separate window at a time. Longitudinal assessment of appointments is performed by inspecting the schedules one day at a time. This is challenging when there are multiple CT simulators and linear accelerators. Similarly, members of the patient care teams wishing to verify the status of documents and important plan parameters associated with their patients, and progress in the creation of the patients' treatment plan may need to open each patient chart in the EMR individually and navigate to the appropriate workspaces. This is cumbersome and prone to omissions.

An internal review by the Department of Radiation Oncology at our institution defined a need for better sharing of information and data across staff and enhanced communication during handoffs among physicians, dosimetrists, medical physicists, and therapists. In addition, there was a need to readily assess resource utilization and progress during the treatment planning process.

In healthcare, a tool used to improve sharing of information, communication, staff awareness, and data quality is the electronic dashboard.^5^ Dashboards were initially developed in the business sector as a system to track key performance indicators and standardized metrics across an organization in an easily understandable visual display for informing decision support.^6^ Central to a dashboard is the ability to reduce complex data into key measures that can be displayed graphically and to integrate data from multiple sources into a single visual display. Increasingly, dashboards are being used in healthcare to provide a concise view of large volumes of data in the form of productivity and quality (performance) indicators to multiple stakeholders at a departmental or organizational level.^7^, ^8^ Quality dashboards visually track performance indicators that guide decisions at the managerial level with the aim of improving practice performance, while clinical dashboards display performance indicators at the patient careteam member level to improve the quality of patient care and clinical workflow.^7^

Assessments of the importance of efforts to track and improve clinical workflow in radiation oncology through dashboards and workflow standardization can be found in the following publications: Sicotte et al.,^9^ customized the EMR to create an automated care pathway‐oriented workflow system that automatically coordinates sequences of activities among the care team. This system was shown to enhance the communication and information flow across staff and to reduce waiting times until a patient's first treatment. Stachelek et al.^10^ have shown that targeted feedback of quantitative data, such as on‐time performance in entering CT simulation orders and in contouring the CT, obtained from a web‐based institutional data repository and an institutional incident learning system, may lead to improved physician compliance thus contributing to improvements in clinical workflow. Targeted feedback was provided in the form of quarterly reports, referred to as dashboards. Agazaryan et al.^11^ implemented a standardized workflow in a Microsoft Access patient database to track the time to complete different activities in the treatment planning process. They also implemented a dashboard in the treatment planning room to communicate CT simulation and projected start dates for patients and the status of the plan as recorded in the patient database. These, combined with continuous process monitoring and modification, were shown to lead to improved timeliness of patient treatments and better quality of care. Separately, in nursing, Wilbanks and Langford ^8^ showed how dashboards combined with databases from electronic health records (EHR) that contain up‐to‐date, comprehensive patient‐centered records could be used for real automated tracking of productivity and quality indicators.

While the above efforts illustrate the usefulness of care coordination and tracking of discrete areas of radiation oncology, there is a lack of a comprehensive, accessible software‐based solution that tracks all aspects of the radiation treatment plan creation and delivery process, ranging from appointments to document status to care path activities, treatment plan status, documents, and parameters for multiple patients simultaneously, based on a standardized workflow, and that also analyses data from these processes to drive practice improvement. Existing commercial solutions are unfortunately not available in every clinic.

The purpose of this study was to implement a comprehensive and interactive, web‐based, EMR‐integrated clinical dashboard to provide real‐time tracking and display of quantitative measures that describe the clinical workflow and resource utilization from the time of CT simulation to the time of treatment delivery for patients receiving radiation therapy.^12^ The dashboard was conceptualized, designed, and implemented by a medical physicist (RM) and was targeted toward dosimetrists, therapists, medical physicists, and physicians. It provides a comprehensive update on workflow and the care of multiple patients at a glance simultaneously by continuously querying the EMR database, processing and displaying the information. This effort entailed implementing a standardized radiation treatment planning workflow within the EMR and providing a consolidated view of all new appointments, documents, and patient care path activities during treatment planning and their status. Since there is a trend for radiation oncology facilities to migrate to cloud‐hosted EMRs, the dashboard was designed to work equally well in real‐time on a locally hosted or a cloud‐hosted EMR.

The dashboard was designed to prospectively gather data that provides insight into how care path activities unfold over time, where delays are introduced, and the effect of introducing new care path activities on clinical workflow.^13^ In this study, we also present preliminary quantitative measures that characterize the treatment planning process including on‐time performance, staff compliance in using the standardized workflow, and how various treatment planning activities unfold over time. In addition, we present preliminary results which quantify the longitudinal effect of COVID‐19 on patient volumes using data obtained from the dashboard. In the long term, we expect this data‐driven effort to contribute to better coordination of care, clinical efficiency, and, consequently, patient safety.

## 2 MATERIALS AND METHODS

The organizational process by which dashboards are developed and implemented in hospital settings can be broken into stages or phases that span from making data inventories to optimizing the dashboards to make them flexible to changes.^14^ In this study, we iterated through the stages defined by the software development life cycle, that is, requirements analysis, planning, software specification, software design, software implementation, testing, and deployment. The software development overlapped with the implementation of a standardized clinical workflow and staff training.

Implementation of the dashboard required a knowledge of web design including web graphic and user interface design, databases, the data dictionary of the EMR, an understanding of the relationships among different fields and tables, Structured Query Language (SQL) programming, client and server‐side coding, server configuration, web‐based security, and radiation therapy workflow. It also required inter‐ and multidisciplinary work including with the institutional information systems division.

Designing and implementing the electronic dashboard involved iterating through the following stages: (i) specification of clinical variables and measures to be tracked, (ii) evaluation of the feasibility of an EMR‐integrated system and alternatives, (iii) specification of the modes of information display on the dashboard, (iv) software specification and implementation for the dashboard, (v) standardizing the clinical workflow and identification of activity control points, (vi) staff training, and (vii) dashboard prototype validation and testing.

Each of these stages is expanded upon in the following subsections.

### 2.1 Specification of clinical variables and measures to be tracked

Relevant variables and measures to be tracked were captured in regular meetings and consultations with radiation therapists, medical physicists, dosimetrists, and physicians. A need to track resource utilization and key care path activities, documents, and treatment‐plan parameters linked to the creation of a patient's treatment plan by date, physician, location, and type of appointment was identified. Appointment type refers to either CT simulation or the type of treatment simulation (simple, complex, stereotactic body radiation therapy (SBRT)). Treatment simulation entails verification that the patient alignment on the linear accelerator is the same as during CT simulation, and the plan is deliverable. The variables to be tracked included:

1. CT simulation and treatment simulation appointment date, time, location, status, and notes,
2. CT simulator and linear accelerator patient load,
3. Due date and status of activities linked to the creation of a patient's radiation therapy treatment plan,
4. Status of documents in a patient's chart,
5. Prescription details and the status of the prescription, radiation treatment plan and beams, and
6. Responsible physician and dosimetrist for a patient's treatment plan.

All staff had access to the same information. Analysis of the above information enabled the derivation of performance measures such as on‐time completion of tasks, machine and staff workload, and total time from CT simulation to treatment.

### 2.2 Evaluating the feasibility and suitability of a stand‐alone system versus an EMR‐integrated system

An important consideration when designing the dashboard was whether to use a stand‐alone system to acquire and store the identified variables or whether to exploit the data collection capability of the departmental EMR. An EMR‐integrated solution was deemed advantageous since it already contained or could be configured to acquire and save the data required without duplication of work by staff. An EMR‐integrated solution also avoided the need for the creation and maintenance of a separate database.

The dashboard was designed to address inherent limitations in the EMR that preclude the data from being displayed in a form that allows for a consolidated and instantaneous evaluation of workflow and care path activity status. Examples of limitations addressed included aggregating information from disparate windows or present in list form in the EMR into a single quantitative, informative value such as percentage progress in completing a treatment plan, and implementation of a sorting algorithm in software to resolve a lack of association between treatment planning activity status and patient appointment date.

### 2.3 Specification of modes of information display and user interaction

A combination of sortable tables, color‐coded due dates, and interactive charts provided effective display and communication of information to staff.

### 2.4 Software specification and implementation

A web‐based application was chosen in order to facilitate ease of access and deployment of the dashboard. RESTful web design methodology was used to develop a secure, interactive, at fixed intervals web‐based application that updates continuously by querying the underlying SQL database of the EMR at fixed interval or upon user query.

### 2.5 Design

Given the challenge of fitting the large number of variables being tracked into a single display, a tabbed display was adopted whereby information in each of the tabs or views was unified by appointment date, location, appointment activity type, and physician name. An important design consideration for the dashboard was the need to present information to the user in a concise and coherent manner. This is challenging given the complexity of the radiation treatment planning process, the large number of personnel involved and the variety of information we wished to consolidate. We sought to capture clinical workflow and resource utilization at multiple scales proceeding programmatically from a broader overview at the machine level to a more detailed view at the patient care path level. The sequence in which the tabs and their contents were ordered reflected the sequence of clinical workflow processes required for the creation of the treatment plans. Furthermore, the dashboard was designed such that the combined view of the individual elements conveyed a top‐level and contextual overview of the different processes being tracked at a glance.

### 2.6 Standardization of clinical workflow and identification of activity control points

The next step in implementing the dashboard consisted of creating a clinical workflow that permitted the display of progress in the creation of a patient's radiotherapy plan. A standardized workflow, suitable for all stakeholders, which could be implemented in the EMR and tracked by the dashboard was formulated. Process maps and flowcharts were created to model clinical workflow from the time of CT simulation to treatment simulation. These are described as follows:

1. Standardized care path activities, their flow, and documentation associated with a patient's care path for various treatment modalities from the time of CT simulation to treatment simulation,
2. Care path activity and document completion sequence, dependencies and timeline,
3. Care path activity and document ownership (responsible parties), and
4. Communication among medical physicists, dosimetrists, physicians, and radiation therapists and handoffs.

The status of activities associated with the creation of the treatment plan was tracked by means of control points in the form of appropriately dated task sets in the EMR. The task sets comprised quality checklist (QCL) items, the formal term used in the EMR to refer to a task, with one QCL per care path activity. The task sets were tailored by treatment type and modality. They each comprised a subset of relevant QCLs to be completed in the EMR. For each patient, the trigger point for generating the appropriate task set was at CT simulation. Five different task sets were created to reflect the different complexities associated with 3D, 3D boost, intensity‐modulated radiotherapy (IMRT), IMRT boost, and SBRT treatment plans. An ideal timeline from the date of CT simulation was assigned to the QCLs in the task sets based on perceived clinical timeline, staffing levels, and departmental throughput. Instructions on a particular activity were entered as comments in the QCLs by the appropriate staff member.

We decided to use QCL task sets to implement the standardized workflow for two reasons. First, generating all QCLs simultaneously with a specific due date according to the ideal timeline served to provide advanced warning to a user as to when a specific task was due. Second, a limitation of the EMR was that the SQL tables do not contain a key to relate individual QCLs associated with the same treatment plan, but which are not part of a task set, to each other or to a specific treatment simulation time. This limitation was addressed by using task sets, since task sets and the QCLs therein are linked by a unique key. This key along with a sorting procedure based on treatment simulation time, and task set creation date and time, were used to associate specific QCLs with a specific treatment plan and treatment simulation time.

Document status was tracked by the dashboard through direct querying of the radiotherapy documents contained in the EMR.

### 2.7 Staff training

The proposed clinical workflow and new task sets were reviewed by the appropriate section leads. Physicians, medical physicists, dosimetrists, and radiation therapists were trained in the generation and completion of the appropriate task sets and QCLs in the EMR, and on how to perform handoffs through the use of comments in the tasks, in accordance with the steps described in flowcharts modeling the clinical workflow. They were also trained in how to use the dashboard to track resource utilization and progress in the creation of a patient's treatment plan. Training was performed through individual or small group meetings with the dashboard users.

### 2.8 Dashboard validation and testing

Testing and incorporation of feedback was performed continuously during the development of the dashboard. Dashboard prototypes of increasing functionality were released verified, and validated iteratively, to ensure the specifications described in Section 2.1 were met, and the information displayed on CT simulations, CT workload, linear accelerator new treatment simulations, linear accelerator workload, task due dates and status, document status, prescription details, and treatment plan status was tractable in real‐time and accurate.

### 2.9 Quantitative measures of workflow

Several performance measures indicating how different treatment planning activities unfold over time were calculated from the data monitored by the dashboard.

At the beginning of the COVID‐19 pandemic, we noticed large fluctuations in the number of CT simulations and treatments displayed on the dashboard. We used this data to quantify the early impact of the first two waves of COVID‐19 on radiation oncology practice. Interrupted time series (ITS) analyses were conducted using electronic health record data spanning January 2018 to April 2021. Segmented regression models were used to estimate the immediate impact of our state's COVID‐19 Wave1 and Wave2 on the daily and weekly number of CT simulations. State‐level COVID‐19 data were used to define the start and end of Wave1 as well as the start of Wave2.

## 3 RESULTS

The purpose of this study was to implement a comprehensive and interactive, web‐based, EMR‐integrated clinical dashboard to provide real‐time tracking and display of quantitative measures that monitor resource utilization and describe how different clinical processes unfold over time.

### 3.1 System architecture

The final version of the dashboard was reached following an iterative process consisting of formulating the dashboard specifications, designing, implementing, verifying, and validating a prototype, presenting the prototype at departmental meetings and further refining the clinical workflow and dashboard specifications based on staff feedback. Throughout this endeavor, we worked with our institutional information systems team to obtain necessary approvals.

**FIGURE 1:**
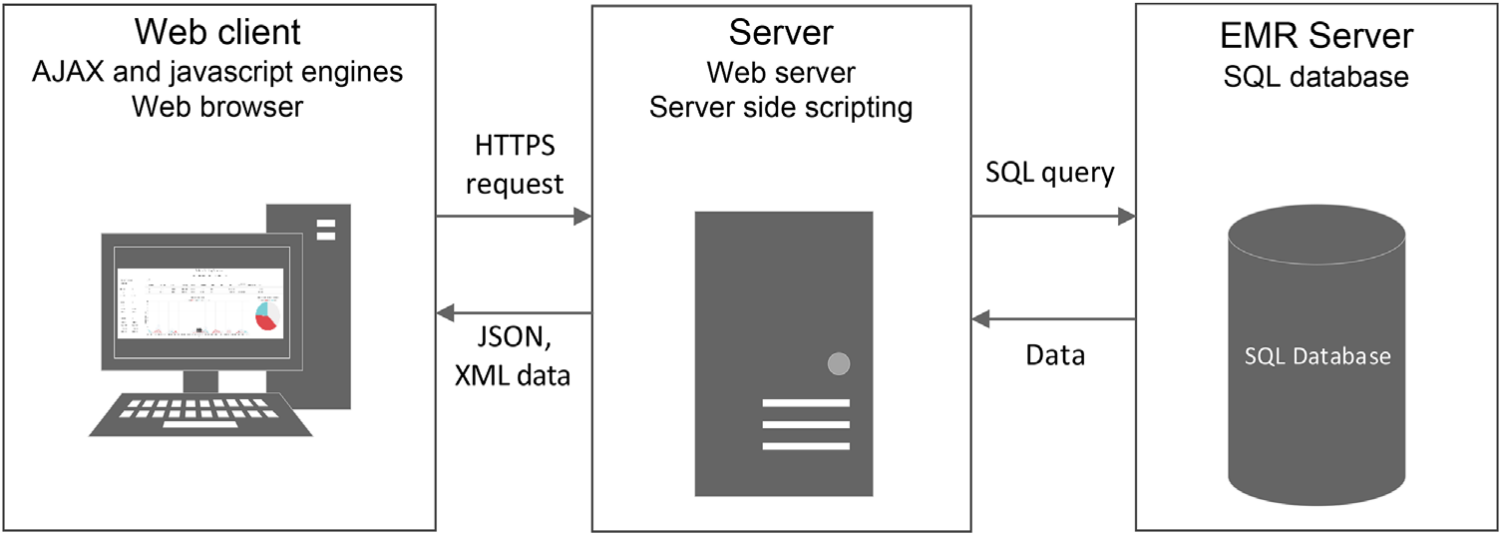
System architecture. The three main components of the dashboard are the web clients, a server, and a SQL database. The server translates queries from the web client into SQL code, which is used to query the database. The results from the SQL queries are processed by the server before being displayed on the dashboard of the web client. All communication is encrypted

The underlying system architecture of the dashboard is shown in Figure 1. It consists of individual web clients, a server, and a SQL database. The dashboard is displayed on the web clients. Queries from the web clients are relayed to the server, which translates them into SQL queries to query the EMR database. The results of the SQL queries are returned as hundreds of rows of data. These are processed by the server and returned to the web clients. Communication between the web clients, server, and SQL database was achieved through Ajax, JSON, and SQL and is encrypted.

The data for the dashboard was obtained from 15 different tables in the EMR SQL database using complex SQL queries. These involved joins across multiple tables with nested subqueries and conditions. Given the complexity of the queries, they were tested continuously throughout the development of the dashboard, to ensure the results were as expected.

The combination of client‐side and server‐side rendering with the use of hypertext transfer protocol secure (HTTPS) and individual authenticated user accounts enhanced the security and performance of the system. The web client constantly updates relevant sections of the webpage in real‐time while avoiding reloading the whole page using javascript. Server‐side SQL querying enhanced security by preventing the web clients from directly accessing the EMR database.

### 3.2 Standardization of workflow and treatment planning timeline

**FIGURE 2:**
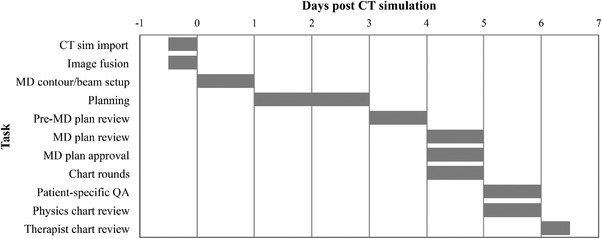
IMRT treatment planning timeline. The Gantt chart displays the care path activities that need to be completed during the creation of an IMRT treatment plan and the timeline associated with each of the activities

Prior to the development of the dashboard, the department did not have a cohesive standardized workflow or process to communicate progress in the creation of a patient's treatment plan. Care path activities and QCLs were introduced to address these shortcomings. Fourteen care path activities, each of which was represented by a QCL in the EMR, were identified to capture the treatment planning process. Five QCL task sets were generated for three treatment modalities, 3D, IMRT, and SBRT). The task sets comprised the QCLs required for initial and boost plans. For each task set, an ideal timeline was assigned to the constituent QCLs in the EMR, with zero being at the end of the day on which the CT is acquired. A Gantt chart of the timeline for care path activities or QCLs comprised in IMRT and 3D initial plans is shown in Figure 2. The number of days shown in the Gantt chart is counted post‐CT simulation as described above. When a task set is generated for a patient, the constituent QCLs are assigned due dates based on the timeline post‐CT simulation.

### 3.3 Radiation oncology dashboard

The dashboard displays information over five views: CT Sims, Linac Sims, Tasks, Documents, and Diagnosis and Interventions, unified by a set of filters consisting of a user‐selected appointment date, appointment location (CT simulator or linear accelerators), clinical activity type (CT simulation, simulation simple, simulation complex, or SBRT), and physician name. The dashboard updates continuously to reflect changes in the EMR. Note that activity type in the dashboard filter differs from the care path activities tracked during the treatment planning process. Activity type here reflects the nomenclature used in the EMR for appointment type.

#### 3.3.1 CT simulations

**FIGURE 3:**
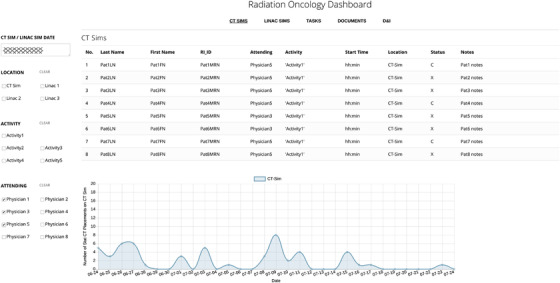
CT simulations. The dashboard consists of five tabs (CT sims, Linac sims, Tasks, Documents, and D&I shown at the top of the panel) unified by a set of filters comprising the date, appointment location, appointment type, and physician. The CT simulations tab displays details of patient appointments on the CT scanner as per the selected set of filters. The graph displays patient load in a period of ± 2 weeks from the selected date

The first view, shown in Figure 3, is labeled CT Sims and provides information on CT simulations according to the selected date and filters. The information displayed in this view was queried from six different tables containing patient, staff, and scheduling information. The top part of the view displays a table listing patient's name and medical record number (MRNs), attending physician's name, type of CT simulation, appointment time, location and status (completed, cancelled), and notes associated with the appointments. The bottom part of the view displays a graph, which shows the number of completed or scheduled appointments in an interval of ± 2 weeks from the selected date. This interval can be adjusted. The graph of the daily view of the CT sim workload helps with longitudinal workload assessment and provides scheduling information for a given physician's patients by appointment activity type, date, and location.

#### 3.3.2 New treatment simulations on linear accelerators

**FIGURE 4:**
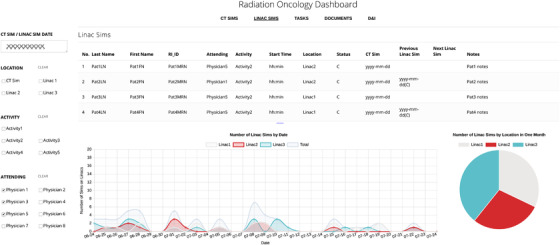
New treatment simulations. The Linac Sims tab provides details of new treatment simulation appointments on the linear accelerators. Contextual information regarding the patient's CT simulation and any previous or future treatment simulation appointments is also provided. The graph provides a longitudinal assessment of patient load distribution on the different linear accelerators for a period of ± 2 weeks surrounding the selected date. The pie chart provides details about the total load on the different linear accelerators over a period of a month centered around the chosen date

The Linac Sims view, shown in Figure 4, provides a summary of new treatment simulations on the linear accelerators. Treatment simulations are performed to validate the deliverability of a planned radiation treatment prior to the first appointment. Best practice recommends that the treatment plan and patient documentation be approved prior to treatment start. This view provides a list of all new treatment simulations on the different linear accelerators for a given date at a glance.

As with the CT sim view, this view displays a table in the top part and charts in the lower part. A summary of patient appointment times, status, and treatment simulation history with respect to new patient treatment simulations on the linear accelerators, CT simulation date and patient notes are shown in a table. Thus, the table, informing the user of all new simulations on the treatment machines in the department at a glance. Contextual information about the current treatment simulation appointment with respect to the CT simulation, previous and future treatment simulations provides a visual assessment of whether the patient has undergone previous radiation therapy or if they are scheduled for an initial or boost treatment.

The linear accelerator color‐coded graph and pie chart shown in Figure 4 display the distribution of new treatment simulations on the linear accelerators by date and over a period of ± 2 weeks from the selected date. The department aims to maintain a balanced distribution of patient treatments on the different linear accelerators by treatment type and complexity, and these charts provide a visual, quantitative display of linear accelerator workload. The graph of treatment simulations by dates provides the feature of advanced notice at a glance of all specialized procedures, such as SBRT treatments, that require the presence of a physician or medical physicist, thus facilitating the timely provision of care.

#### 3.3.3 Tasks

**FIGURE 5:**
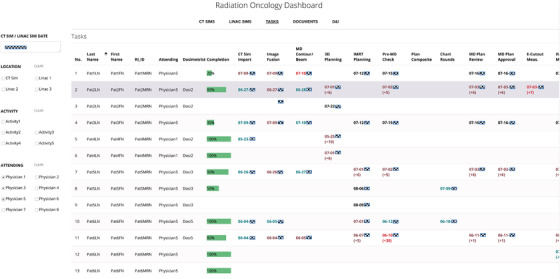
Tasks. The tab displays the status and due dates of the QCLs associated with the creation of a patient's treatment plan for all patients whose treatment is due to start on the selected date. The due dates are color coded with black indicating a pending but on time task, green indicates on time completion, brown indicates late completion, and red indicates an overdue task. Early or late completion is also indicated by means of the appropriate number of days in brackets next to the due date. Overall plan completion status for every patient is indicated by means of a labeled progress bar

Data for the tasks view is queried and consolidated from seven different tables in the SQL database. The tables contain patient, scheduling, task set, QCL, and staff information. The tasks view is displayed in Figure 5. It provides a quantitative and visual display of progression in the care path activities associated with a radiotherapy plan from the time of CT to treatment simulation by patient and thus addresses a major limitation of the EMR, which does not have this capability. Color‐coded due dates and statuses permit users to assess how the clinical workflow associated with a patient's treatment plan unfolds with time.

For every patient, the QCL task sets are generated in the EMR at CT simulation by the therapists according to the simulation order entered by the physician, and the QCLs therein are automatically assigned a due date as shown in Figure 5 and responsible party.

The displayed due dates are color coded, with black indicating a pending, on‐time task, green representing on‐time completion of the task, brown representing late completion, and red representing an incomplete task. The number of days by which a task is overdue, if incomplete or completed late, or the number of days in advance that a task was completed is listed in brackets by the color‐coded due date. The horizontal green bar on each row is a numerically annotated progress bar that displays the percentage of all QCLs completed for a particular treatment plan and provides a quantitative measure of overall readiness of a plan for treatment.

The table also lists the attending physician and dosimetrist assigned to a particular treatment, appointment time and location for the patient's treatment simulation.

#### 3.3.4 Documents

**FIGURE 6:**
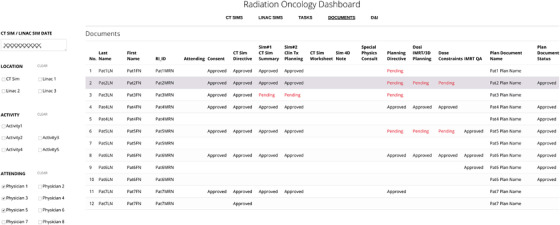
Documents. The Documents tab lists the approval date (shown here as “Approved” for deidentification purposes) of all documents in a patient's treatment chart for patients with a CT simulation or treatment simulation on the selected date. If the documents have not been approved, their status is listed. Unapproved, pending documents are highlighted in red. All documents require approval prior to the start of treatment

At different stages of the treatment planning process, a number of documents are completed and approved by the patient's care team in the EMR. All relevant documents are required to be completed prior to the start of a patient's CT simulation or treatment simulation. Verifying the status of the documents in the EMR requires the user to manually open the chart of every patient individually and to browse through the documents workspace. The Documents tab, displayed in Figure 6, consolidates information from seven tables in the SQL database to provide a summary of the status of all documents in a patient's treatment chart. Negatively framed tables were used with unapproved, pending documents highlighted in red. If approved, the date of approval is displayed in black. For the purposes of deidentifying the data, we have replaced the date, that would normally be displayed, with approved in Figure 6. This view provides advanced notification of any documents that are missing or pending approval prior to these procedures for multiple patients simultaneously.

#### 3.3.5 Diagnoses and interventions

The Diagnoses and Interventions view, not illustrated, displays a table of patients along with their prescription information and parameters that are central to their treatment requiring verification prior to treatment. These include the prescription dose and physician‐approval status, radiation treatment beam approval by a medical physicist, whether the imaging isocenter for the cone beam CT images to be acquired during treatment has been specified, if a patient is on a protocol and special instructions for the treatment. In the EMR, this information is spread across a number of different windows in the Diagnoses and Interventions workspace, which are cumbersome to navigate to and which not all users may be familiar with. An integrated view of this information on the dashboard now allows users to instantly assess these parameters. This has facilitated timely identification of any potential issues that may result in treatment delays or interruptions.

Data displayed in this view was queried and aggregated from 10 different tables in the EMR SQL database including the patient care plan, site setup, and treatment field tables. Negatively framed tables were used here as well in the dashboard to convey issues with the treatment plan.

### 3.4 Deployment of dashboard

Staff were trained in the completion of the QCLs in the EMR and in how to use the dashboard. The dashboard was initially deployed to the head therapist and a limited number of physicists and physicians prior to being made available to a wider group in the treatment planning area. Since the proposed clinical workflow was new for the department, physicians were enrolled incrementally in the use of the new task sets for their patients. This approach allowed us to resolve issues associated with the new workflow prior to enrolling additional physicians. Improvements made to the QCL‐based workflow after the initial release included reconfiguring the QCLs and designing views in the EMR so that the physicians and dosimetrists would readily know which QCLs had been assigned to them for completion.

### 3.5 Measures of performance of radiation treatment planning workflow

We analyzed data collected for 129 patients and 669 QCLs collected in the first deployment phase of the standardized QCL workflow. The compliance rate in recording completion of the QCLs, average and standard deviation of the number of days elapsed from the day of CT simulation to record of QCL completion, and on‐time performance in recording completion of the QCLs with respect to the ideal timeline are shown in Table 1.

**TABLE 1:** Radiation treatment planning performance measures

	3D				IMRT			
	Compliance	Mean	Std. dev.	On‐time	Compliance	Mean	Std. dev.	On‐time
Task	(%)	(days)	(days)	(%)	(%)	(days)	(days)	(%)
CT sim import	100	−0.36	0.27	91.1	100	−0.34	0.34	90.0
Image fusion	82.3	0.43	1.07	40.0	90	0.45	0.71	28.9
MD contourcontour / beam setup	96.2	0.36	0.88	85.5	98	1.21	0.89	44.9
Planning	82.3	3.44	2.34	41.5	64	5.81	1.76	3.12
MD plan review	34.0	3.22	2.16	86.7	24	4.19	1.97	75.0
MD plan approval	35.4	3.22	2.21	85.7	24	4.17	2.0	75.0
Patient‐specific QA					56	5.98	2.05	32.1
Physics chart review	64.6	3.90	2.31	86.3	60	6.11	1.63	50.0

*Note*: Staff compliance in recording QCL completion in the EMR, the average and standard deviation in the number of days post‐CT sim to complete the QCL, and on‐time performance in completing the QCL with respect to the ideal timeline are shown for 3D and IMRT treatments.

Abbreviations: 3D, three‐dimensional; EMR, electronic medical records; IMRT, intensity‐modulated radiotherapy; QCL, quality checklist, MD: Doctor of Medicine, QA: quality assurance.

The time to completion of the physics chart review indicates the time from CT simulation to approval of the plan for treatment. On average, the physics chart review QCL was completed in the EMR within 3.9 days from the date of CT simulation for 3D treatments, which was significantly less (*p* < 0.05) than the ideal timeline of 6 days. For IMRT treatments, the physics chart review QCL was completed in the EMR within 6.1 days of CT simulation, which was not significantly different from the ideal timeline of 6 days.

Compliance in recording QCL completion ranged from 24% for the MD plan review and MD plan approval tasks to 100% for CT import. The low compliance for the MD plan review and plan approval tasks was addressed by discussing with the staff concerned and making adjustments to address gaps that hindered completion of the tasks. These included its creating physician‐specific views in the EMR for the physicians to easily access the QCLs assigned to them for completion.

The low compliance for the physics and patient‐specific QA tasks was due to the physicists completing separate QCLs generated for these tasks by the dosimetrists in accordance with the previous workflow. This glitch in the workflow was addressed by retraining the dosimetrists to not generate QCLs outside those generated for the standardized workflow.

On‐time performance in recording task completion ranged from 3.1% for planning for IMRT treatments to 91.1% for CT import. The low on‐time compliance for the planning tasks was due to this task being completed out of sequence. That is, it was not being completed prior to the MD plan review task, as per the standardized workflow, but after the MD plan approval task. We noted from this out‐of‐sequence task completion that the medical physicists tended to receive the IMRT plans for plan review later than according to the ideal timeline, for instance, on average 5.8 days after CT simulation as opposed to 5 days, resulting in an on‐time performance of 50% for the physics chart review

### 3.6 Quantitative evaluation of the effect of COVID‐19 on patient volumes

**FIGURE 7:**
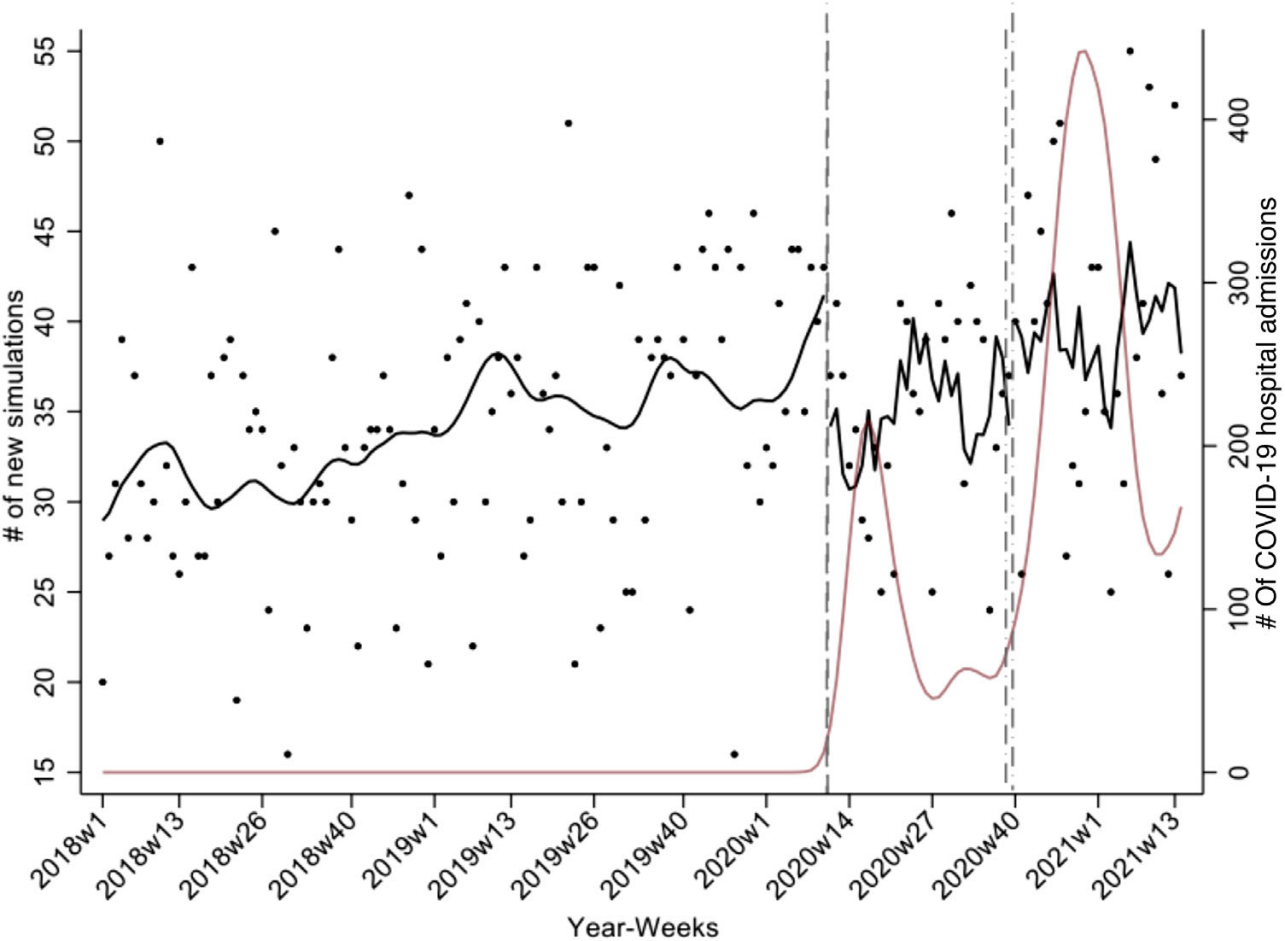
CT simulation case load. The results of an interrupted time series analysis of the effect of COVID‐19 on the weekly number of CT simulations are shown in black. The number of COVID‐19 hospital admissions in the state is shown in dark red

Figure 7 shows the results of the ITS of the effect of COVID‐19 on the number of weekly CT simulations (in black) through the first quarter of 2021. The number of COVID‐19 hospital admissions in the state is shown in red. While an increasing trend in the number of CT simulations was observed from 2018 to 2019, this was not the case in 2020. We observed fewer than usual CT simulations during the first COVID‐19 wave in 2020. After accounting for autocorrelation and seasonality, upon the start of the first wave, an estimated 11.1% fewer simulations (95% CI: −20%, −2%) than would be expected in the absence of COVID‐19 were performed. After the first wave, the CT simulation workload increased by 6% (95% CI: −10%, 22%). Upon the start of the second wave, an estimated 4.8% fewer simulations than would be expected (95% CI: −16%, 7.1%) were performed. As of 11 April 2021, relative to what would be expected in the absence of the pandemic, an estimated, 15.5% fewer CT simulations were performed than were expected (95% CI: −30%, −1%).

**FIGURE 8:**
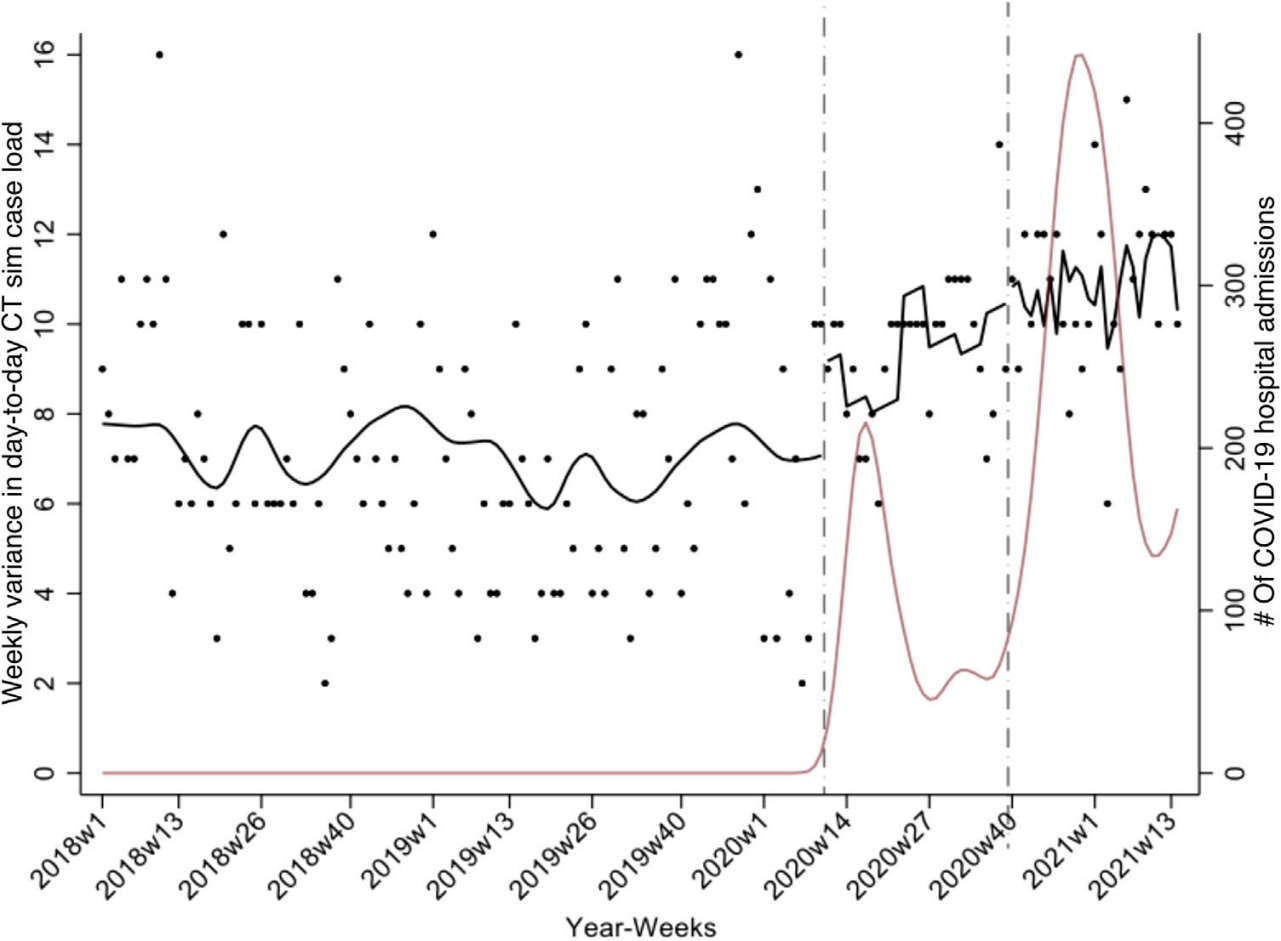
Weekly variance in the day‐to‐day CT simulation case load. The results of an interrupted time series analysis of the effect of COVID‐19 on the weekly variance in the daily number of CT simulations are shown in black. The number of COVID‐19 hospital admissions in the state is shown in dark red

An increase in the weekly variance in the daily number of CT simulations performed was observed during the pandemic. Figure 8 shows the results of the ITS on the weekly variance in the day‐to‐day CT simulation case load. The number of COVID‐19 hospital admissions in the state is shown in red. After accounting for autocorrelation and seasonality, upon the start of the first wave, the weekly variance in day‐to‐day CT simulation caseload increased by an estimated 30.6% (95% CI: −1.2%, 62.4%) relative to what would be expected in the absence of COVID‐19. Over the course of the first wave, the variance in CT simulation case load increased by an estimated 21.5% (95% CI: −14.4%, 57.4%). Upon the start of the second wave, the variance in the number of CT simulations was 58% greater than would be expected in the absence of COVID‐19 (95% CI: 8.9%,1.1%). As of 11 April 2021, relative to what would be expected in the absence of the pandemic, there was an estimated 96.5% increase in the variance day‐to‐day for CT simulations (95% CI: 33.4, 100%).

## 4 DISCUSSION

We have implemented a web‐based dashboard to track clinical workflow and resource utilization in radiation oncology. The dashboard dynamically integrates data queried in real‐time from the departmental EMR and tracks the clinical workflow associated with the radiation treatment planning process for cancer patients over multiple views and multiple scales unified by date, appointment location, type of CT simulation or treatment simulation, and physician name. For a given treatment date, the CT Sims view summarizes imaging appointments and CT load, the Linac Sims view summarizes patient appointment information on the linear accelerators, patient setup history, and linear accelerator load; the Task status view lists the clinical tasks associated with a treatment plan for the patients being imaged or treated on the selected date, the due date of the tasks, status, percentage progress in task completion, and patient appointment details; the Documents view lists the status of documents in the patients' charts; and the Diagnoses and Interventions view summarizes prescription information, imaging instructions, and whether the plan was approved for treatment by a medical physicist.

An incremental approach to deploying the dashboard in the clinic was favored, whereby the dashboard was first released to a limited number of users and physicians were incrementally enrolled in adopting the new clinical workflow. Being web‐based, the dashboard is easily accessible on the institution's intranet. By displaying the same information to all staff, the dashboard facilitates care coordination and handoffs while also improving transparency and information sharing. The efforts described here, along with methods we have developed to formalize and automate quality assurance processes in radiation oncology,^15^, ^16^ are part of a larger project to automate cancer care and improve clinical practice in radiation oncology.

Dashboards have been used for a variety of purposes. Specific areas of application of quality and clinical dashboards in healthcare range from nursing to the emergency room to cancer care. Examples of dashboards employed to improve clinical decision making include work by Liu et al.^17^ who employed a dashboard as a clinical decision support tool for colorectal cancer risk assessment and management, Bersani et al.^18^ who used a dashboard to improve quality and safety in the clinic and, by Dolan et al.,^19^ as a patient decision aid in the selection of nonopioid pain medication. Applications designed to enhance communication of data among healthcare staff include the display of anesthesia records aggregated from multiple databases for children undergoing radiation therapy and who require general anesthesia by Nelson et al.,^20^ improving the visibility of patient data for nurses in computerized physician order entry systems by Tan et al.^21^ and as a tool to visualize multiple patient histories simultaneously by Bernard et al.^22^ Dashboards have also been used to monitor and evaluate performance indicators. Martinez et al.^23^ monitored patient flow and communicate key performance indicators such as length of stay, and operating room delays calculated from EMR‐extracted data to hospital stakeholders using the Donebian model. Curtright et al.^24^ and Mick et al.^25^ proposed a dashboard for performance measurement in healthcare. Stattin et al.^26^ displayed quality of care indicators for cancer patients. Stone‐Griffith et al.^27^ employed a dashboard to increase the effectiveness and efficiency of emergency care, and Randell et al.^28^ developed an interactive tool to evaluate the quality of data collected in national clinical audits. In nursing, dashboards have been used to guide clinical care and practice from EHR‐queried data.^8^

Visualizing large amounts of data along with data integration^6^ is an important part of dashboards. The solution presented in this study combines information for multiple patients and multiple resources into a single, easily interpretable view by processing and integrating the results of multiple SQL queries to the EMR. As patient volumes increase, treatments become more complex, and departmental workload and the amount of data available increase, a consolidated view of pertinent information, such as the one provided in the dashboard, becomes more important and may contribute to improved clinical efficiency and patient safety.

Graphical linguistics have been shown to reduce the cognitive effort for processing and assimilating high‐density time‐sensitive information displayed on dashboards.^29^ In this study, we employed carefully selected visualization and reporting techniques such as tables with sortable columns, graphs, pie charts, progress bars, and color coding^30^ to enhance communication of condensed information to a patient's interdisciplinary care team in real‐time. Simultaneously generated due dates at the time of CT simulation were thought to be more informative and effective than icons^31^ or triggered tasks at conveying the timeline associated with a patient's treatment planning process. In addition to not conveying the entire timeline associated with a plan, a limitation of triggered tasks is that they require 100% compliance from staff in recording care path activity completion, which our study suggests is challenging in clinical practice. Negatively framed tables conveyed the status of documents, treatment prescriptions, and whether a treatment plan was approved for treatment by a medical physicist. In negatively framed tables, overdue or incomplete items are displayed in red and no color coding is employed for completed items and may lead to more correct decision‐making than positively framed tables.^31^

The system integrates features from both quality and clinical dashboards.^7^ In addition to allowing users to quickly assess progress in a patient's care path, the ability for physicians to view patient appointment and care path details in a consolidated manner, in real‐time may facilitate clinician decision‐making particularly, with respect to adjusting staffing levels during periods of high anticipated workloads and with load balancing on the different machines. It has been observed that increases in departmental workload in radiation oncology may lead to an increase in errors and incidents.^32^ Separately, through failure mode and effects analysis, the CT simulation and treatment planning stages have been identified as being the main causes of delays in the initiation of treatment in radiation oncology.^33^ Such studies highlight the need for data‐driven approaches that help when developing targeted approaches to improve safety.

Dashboards not only facilitate immediate decision‐making but may also serve as a learning tool.^6^ We have presented results that now make it possible to document the time required to complete the different care path activities and compliance in using the QCL task set generated for the dashboard. In the future, we plan to examine if compliance and on‐time QCL completion improved with time. We also presented preliminary results that showed the effect of the first two waves of COVID‐19 on patient volumes. Despite radiation oncology facilities remaining fully operational during the pandemic, large fluctuations in patient volumes and workload were observed. Interestingly, while a decrease in patient volumes was observed relative to what would be expected in the absence of COVID‐19 during the first and second waves, an increase in variance in the day‐to‐day number of CT simulations was observed. In the presence of fixed timelines, such variations constitute a strain on clinical resources. Fixed timelines, however, may not be maintainable, and such large fluctuations could result in delays in treatment starts. We now plan to quantify these effects. Our findings may help inform preparations for future novel aberrations in the workflow.

Implementation of the dashboard has helped improve and standardize clinical workflow in the department. It has also provided the operations data necessary for further refinement of the standardized clinical workflow. During the initial rollout of the dashboard and afterwards, we reviewed the data acquired carefully and made a number of interventions to ensure the use of the standardized QCL workflow and dashboard as intended. Changes in the workflow were implemented in such a way that there was minimal disruption of existing practice. Adherence to the workflow was assessed quantitatively from data queried from the EMR. However, retraining, emphasis on teamwork, and a period of adjustment were required to ensure adoption of the new workflow. Our data‐driven change implementation process may serve as a valuable lesson to any clinic that is trying to adopt a new standardized workflow that can be tracked. Possible improvements to the dashboard include the ability to display practice information for a range of dates, sending reminders to responsible parties when an activity is due, and automatically generating data analytic reports. The main purpose of the dashboard was to provide real‐time display and tracking of clinical processes. However, this may be limiting for users who wish to modify information linked to a patient's treatment directly from the dashboard. In such a scenario, any risks associated with writing to the EMR database outside of the EMR should be considered carefully.

Our efforts to obtain quantitative measures describing clinical workflow are in accordance with best practices to improve patient safety in radiation oncology.^34^ We anticipate these quantitative measures will help identify further areas of improvement in the clinic and enable informed decision‐making with respect to workflow changes, staffing levels, and resource availability. Overall, we anticipate that advanced and effective communication of bottlenecks and issues in the radiation treatment planning process will permit their resolution in a safe and timely manner. We expect the ability to visualize and analyze real‐time health information will contribute to improvements in time from patient consult to first treatment while ensuring patient safety. In future work, we plan to quantify the impact of the dashboard on clinical efficiency and plan quality. The dashboard can be adapted for other clinical practices that use EMR data systems.

## 5 CONCLUSIONS

We have developed an interactive, EMR‐integrated, web‐based dashboard with the aim of improving communication, information sharing, clinical efficiency, and patient safety in a modern and busy radiation oncology clinic. This study indicates that it is feasible to develop and implement a dashboard tailored to the needs of an interdisciplinary team, and which derives and integrates information from multiple tables in the EMR database, for real‐time display of clinical workflow and analysis of performance measures in radiation oncology.

## CONFLICT OF INTEREST

The authors declare no conflicts of interest.

## AUTHOR CONTRIBUTION

R.M.: provided the conception and design of the study, design, implementation, testing and deployment of the dashboard, design and implementation of the clinical workflow, staff training, expert advice and data acquisition, analysis, and interpretation, drafting of the manuscript, and critical revision for important intellectual content, and T.M.R.: provided the conception, design, and implementation of the clinical workflow, review and validation of the dashboard, and critical revision of the manuscript; K.L.L.: validation of the clinical workflow, provision of data, testing of the dashboard, expert advice on the COVID19 study and data analysis, and critical revision of the manuscript; R.C.C.: provided advice and assistance with the implementation of the web‐based components of the software implementation, critical revision of the manuscript for submission; U.S.: provided expert advice on the COVID‐19 study and analysis of the data, and critical revision of the manuscript; SA: provided expert advice, performed statistical analysis, and drafted results for COVID‐19 data, and critical revision of the manuscript; M.G.: provided expert advice on the COVID‐19 study and data, and critical revision of the manuscript; G.L: provided expert advice, support and assistance with the implementation of the web‐based components of the software, and critical revision of the manuscript; E.E.K.: provided the conception for the clinical dashboard, reviewed the dashboard, revised the manuscript critically for important intellectual content.
